# Application of low-intensity pulsed ultrasound on tissue resident stem cells: Potential for ophthalmic diseases

**DOI:** 10.3389/fendo.2023.1153793

**Published:** 2023-03-17

**Authors:** Zichun Lin, Liyu Gao, Ning Hou, Xushuang Zhi, Yupeng Zhang, Zelin Che, Aijun Deng

**Affiliations:** Affiliated Hospital of Weifang Medical University, School of Clinical Medicine, Weifang Medical University, Weifang, SD, China

**Keywords:** low-intensity pulsed ultrasound (LIPUS), stem cells, tissue endogenous stem cells, ophthalmic diseases, clinical application, mechanism, treatment

## Abstract

**Introduction:**

Tissue-resident stem cells (TRSCs) have the ability to self-renew and differentiate throughout an individual’s lifespan, and they utilize both mechanisms to maintain homeostasis and regenerate damaged tissues. Several studies suggest that these stem cells can serve as a potential source for cell-replacement-based therapy by promoting differentiation or expansion. In recent years, low-intensity pulsed ultrasound (LIPUS) has been demonstrated to effectively stimulate stem cell proliferation and differentiation, promote tissue regeneration, and inhibit inflammatory responses.

**Aims:**

To present a comprehensive overview of current application and mechanism of LIPUS on tissue resident stem cells.

**Methods:**

We searched PubMed, Web of Science for articles on the effects of LIPUS on tissue resident stem cells and its application.

**Results:**

The LIPUS could modulate cellular activities such as cell viability, proliferation and differentiation of tissue resident stem cells and related cells through various cellular signaling pathways. Currently, LIPUS, as the main therapeutic ultrasound, is being widely used in the treatment of preclinical and clinical diseases.

**Conclusion:**

The stem cell research is the hot topic in the biological science, while in recent years, increasing evidence has shown that TRSCs are good targets for LIPUS-regulated regenerative medicine. LIPUS may be a novel and valuable therapeutic approach for the treatment of ophthalmic diseases. How to further improve its efficiency and accuracy, as well as the biological mechanism therein, will be the focus of future research.

## 1 Introduction

Stem cells are categorized based on their ability to differentiate into pluripotent, multipotent, and unipotent stem cells, with the latter also known as adult stem cells. Pluripotent stem cells include embryonic stem cells and induced pluripotent stem cells (1). Adult stem cells have a limited differentiation capacity but play a crucial role in tissue regeneration, as they are the endogenous stem cells in the tissue almost always.

**Figure 1:**
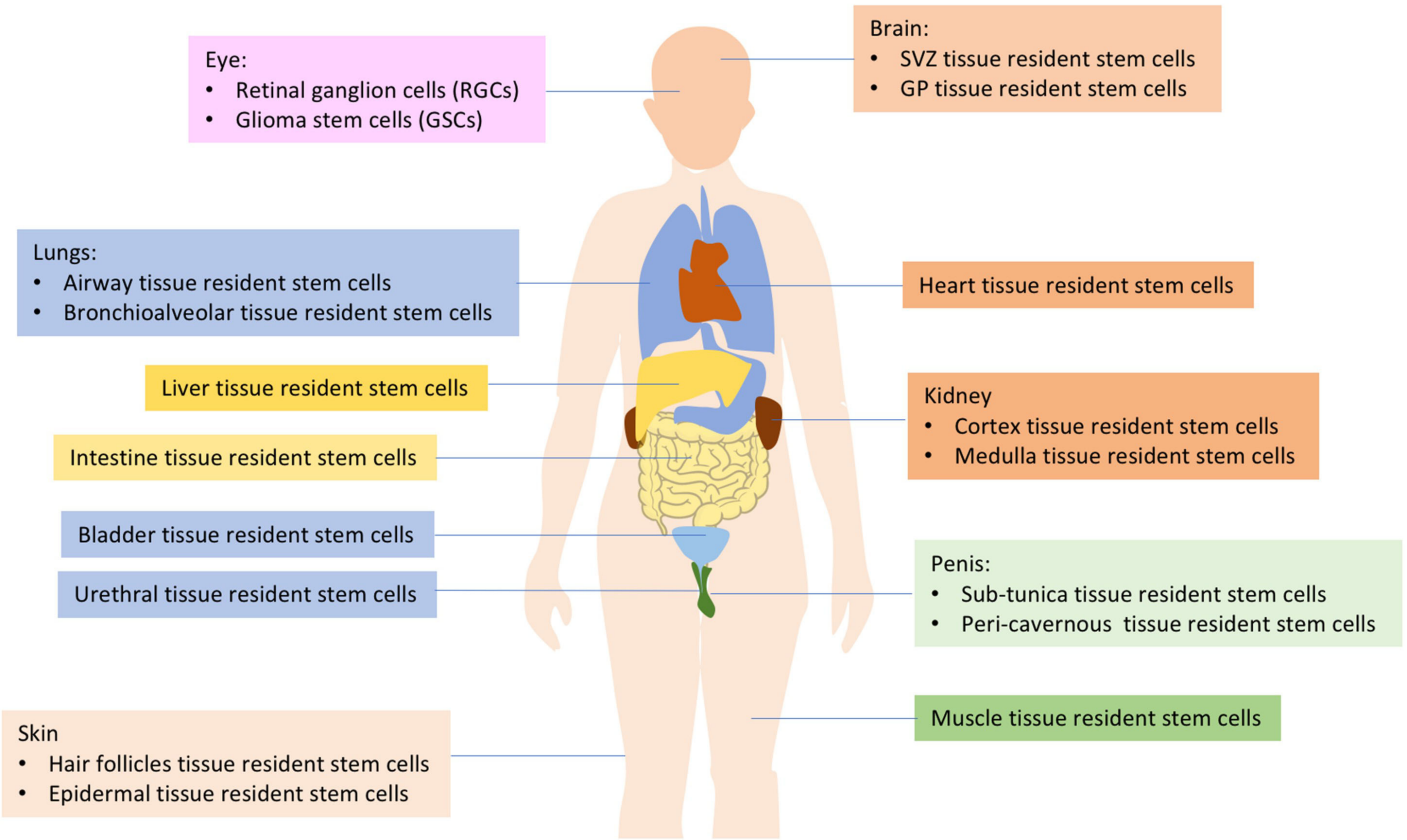
Distribution of tissue-resident stem cells (TRSCs).

The human body is comprised of various cell types, and the turnover rates of the cell types that construct each organ vary. As many as 10^11^ cells die in each adult every day (2). Rapidly turning over cells are replaced by the progeny of highly active stem cells, often referred to as “tissue-resident stem cells (TRSCs)” or “adult stem cells” (1, 3). These TRSCs play a critical role in maintaining tissue homeostasis and repairing injuries, which is dependent on interactions with stem cell niches (3, 4). Current research has identified TRSCs in important organs such as the brain, eyes, lung, heart, kidney, urethra, penis, bladder, muscle, and more (as shown in **Figure 1**). These cells are capable of self-renewal and differentiation throughout an individual’s lifespan, utilizing both mechanisms to maintain homeostasis and regenerate damaged tissues (4–6). It has been suggested that tissue-resident adult stem/progenitor cells offer the potential for *in vivo* differentiation stimulation or using their *ex vivo* expanded progenies for cell replacement-based therapies with multiple applications in humans (5). Such applications include Parkinson’s and Alzheimer’s diseases, Type 1 or 2 diabetes mellitus, as well as eye, liver, lung, skin, cardiovascular disorders, aggressive and metastatic cancers (4, 5). Each type of tissue resident stem cell has its own unique characteristics and functions, making them important for maintaining tissue homeostasis and regeneration (**Table 1**).

**Table 1:** Tissue resident stem cells’ characteristics.

Tissue resident stem cells	Characteristics
Hematopoietic stem cells (HSCs)	These are found in the bone marrow and give rise to all blood cell types. HSCs are highly self-renewing and are able to differentiate into mature blood cells including red blood cells, white blood cells, and platelets.
Mesenchymal stem cells (MSCs)	These are found in many tissues including bone marrow, adipose tissue, and umbilical cord blood. MSCs have the ability to differentiate into bone, cartilage, fat, and muscle cells. They also have anti-inflammatory and immunomodulatory properties that make them promising candidates for cell-based therapies.
Neural stem cells (NSCs)	These are found in the brain and spinal cord, and are responsible for the growth and maintenance of neurons and glial cells. NSCs have the ability to differentiate into neurons, astrocytes, and oligodendrocytes.
Epithelial stem cells (EpiSCs)	These are found in epithelial tissues such as the skin and intestine, and are responsible for tissue repair and regeneration. EpiSCs can differentiate into various types of epithelial cells and help maintain the structural integrity of the tissue.
Endothelial stem cells (EnSCs)	These are found in the blood vessels and are involved in the maintenance of vascular health. EnSCs are capable of differentiating into endothelial cells, which line the interior of blood vessels, and play an important role in vascular repair and regeneration.
Muscle stem cells (MuSCs)	These are found in skeletal muscle tissue and are responsible for the regeneration of muscle fibers. MuSCs are able to differentiate into myoblasts which fuse together to form mature muscle fibers.

Low-intensity pulsed ultrasound (LIPUS) has recently been shown to be a novel approach for modulating stem cells. LIPUS is a specific type of therapeutic ultrasound that operates at low intensity and uses pulsed waves (7). Therapeutic ultrasound has the advantage of producing controlled biological effects noninvasively (8). The use of ultrasonic energy for therapy has continued to expand, with approved applications now including uterine fibroid ablation, cataract removal (phacoemulsification), surgical tissue cutting and hemostasis, transdermal drug delivery, and bone fracture healing, among others (9). LIPUS has minimal thermal effects while still delivering acoustic energy to the target tissue, allowing for noninvasive physical stimulation for therapeutic applications (10). The beneficial biological effects of LIPUS are the result of mechanotransduction, producing microturbulence within the intercellular and intracellular fluids in the vicinity of the wave (11).

Currently, LIPUS has been successfully applied in clinical practices to promote fracture healing, accelerate soft tissue regeneration, and inhibit inflammatory responses. It has also become a tool for enhancing regeneration and tissue engineering by modulating tissue-resident stem cells. Recently, researchers discovered that LIPUS protects retinal ganglion cells (RGCs) from apoptosis and axonal atrophy after optic nerve crush injury and also protects the retina from degenerative thinning (12).

This review presents an overview of the research progress in the tissue resident stem cells, biological effects of LIPUS on stem cell, molecular signaling pathway, preclinical and clinical applications of LIPUS.

## 2 Effects of LIPUS on tissue resident stem cells and related cells

LIPUS is a non-invasive medical treatment that uses high-frequency sound waves to stimulate tissue healing and regeneration, proving to be a novel approach to modulate TRSCs. During LIPUS treatment, a small handheld device is placed on the skin over the affected area. The device emits high-frequency sound waves that penetrate the tissues and stimulate cellular activity. The sound waves cause microscopic vibrations in the tissues, which activate cell signaling pathways and promote the production of growth factors and other molecules involved in tissue repair and regeneration (7, 10, 11).

### 2.1 Key parameters of LIPUS

Some of the key parameter (13, 14) that can be adjusted to optimize the effectiveness of LIPUS for stem cells including: 1). Frequency: LIPUS typically uses frequencies in the range of 20 kHz to 1 MHz, but the optimal frequency can depend on the specific application and the tissue type. For example, bone tissue may respond best to frequencies in the range of 20-40 kHz, while cartilage tissue may respond best to frequencies in the range of 100-500 kHz. 2). Intensity: LIPUS can be delivered at different intensities, ranging from very low intensities (typically < 10 mW/cm^2^) to higher intensities (up to several hundred mW/cm^2^). The optimal intensity can depend on the specific application and the tissue type. For example, bone tissue may require higher intensities to achieve the desired effects, while cartilage tissue may respond better to lower intensities. 3). Duration: The duration of LIPUS exposure can also vary depending on the specific application and the tissue type. In general, longer exposure times may be required for tissues with lower metabolic rates or slower rates of cell proliferation. 4). Duty cycle: The duty cycle of LIPUS refers to the percentage of time that the ultrasound is on versus off. Duty cycles can range from continuous (100% on) to pulsed (less than 100% on). Pulsed duty cycles are often used in LIPUS applications for stem cells, as they have been shown to be more effective than continuous duty cycles. 5). Spatial distribution: The spatial distribution of LIPUS can also be important, as it can affect the distribution of acoustic pressure and the resulting effects on the tissue. This can be controlled by the size and shape of the ultrasound transducer, as well as the positioning of the transducer relative to the tissue being treated.

### 2.2 Ophthalmic tissue resident stem cells

Stem cells have potential therapeutic applications in ophthalmology due to their ability to regenerate damaged tissues and repair the eye. There are several types of stem cells that can be used in ophthalmology, including corneal epithelial stem cells, retinal pigment epithelium stem cells, and mesenchymal stem cells. Some potential therapeutic applications of stem cells in ophthalmology include corneal repair, retinal repair, glaucoma treatment, gene therapy and drug development.

Recently, studies have demonstrated the presence of several endogenous stem cell populations in multiple sites of the eye, which play a role in many eye diseases (15). Limbal stem cells exist in both the inner and outer limbus and are believed to generate short-term progenitor cells that can move concentrically to the central cornea or delaminate (16). Stem cells in the Schwalbe lineage region may constitute a population of adult stem cells with the ability to compensate for the loss of trabecular meshwork (TM) and/or corneal endothelial cells (17). Corneal stem cells are also present and maintain the integrity of the corneal epithelium, protect it from external physical or chemical damage, prevent invasion and damage from pathogenic microorganisms, and inhibit immune inflammation and corneal neovascularization, thereby maintaining the stability of the ocular surface.

Postnatal iris pigment epithelium (IPE) cells are characterized by neural stem/progenitor cells that can proliferate efficiently and exhibit remarkable plasticity. Under appropriate conditions, IPE can generate a variety of cell types, including retina-specific neurons and glia, and lenses (18). The ciliary body (CB) and retinal pigment epithelium (RPE) also contain pigment cells with neuronal progenitor-like characteristics (19). These stem cell populations offer new potential sources for basic stem cell biology and therapeutic applications in retinal diseases.

Trabecular meshwork stem cells (TMSCs) are homogeneous and pluripotent, with the ability to differentiate into phagocytic TM cells. These cells may be able to repair damaged trabecular meshwork and restore functional regulation of aqueous outflow, offering potential for novel stem cell-based therapies for glaucoma (15, 20, 21). Additionally, retinal Muller glia were recently discovered to differentiate into retinal ganglion cells (RGCs), providing a local source of endogenous cells for replacement therapy. Traumatic optic nerve (ON) degeneration and glaucoma-induced retinal ganglion cell death pose major challenges to vision loss. Therefore, preventing RGC apoptosis and promoting regeneration may provide opportunities for the treatment of glaucoma and other optic neuropathies (9, 22).

#### 2.2.1 Effects of LIPUS on Glioma stem cells

Glioma stem cells (GSCs) are a type of cells resembling neural stem cells, with strong self-renewal, unlimited proliferation, and multi-directional differentiation potential. Song et al. (23) investigated the effect of LIPUS on GSCs by inducing glioblastoma cell-derived GSCs *in vitro* and stimulating them with LIPUS for three consecutive days. After measuring relevant markers on the cell surface, the researchers found that LIPUS can induce GSCs to lose stem cell-related characteristics and also “awaken” dormant GSCs from the quiescent G0 phase to the active G2/M phase, thus inducing GSC differentiation. Considering the clinical significance of GSCs, the researchers also observed that LIPUS can increase the sensitivity of GCSs to the chemotherapy drug temozolomide (TMZ) and reduce drug resistance compared to the blank control group.

#### 2.2.2 Effects of LIPUS on Retinal ganglion cells

In ophthalmic diseases such as glaucoma and traumatic optic neuropathy, Retinal Ganglion Cells (RGCs) injury and apoptosis are significant factors affecting visual acuity in patients. Previous studies demonstrated that ultrasound can stimulate the retina of mice and rapidly modulate visual sensitivity (24). Recently, Zhou et al. (12) used LIPUS in optic nerve compression animal models and *in vitro* RGCs degeneration models to investigate the regulatory effect of LIPUS on retinal tissue-resident stem cells. Low-intensity LIPUS (Grade 1 and 2) was found to improve RGCs’ cell viability and prevent apoptosis in optic nerve compression. The optimal LIPUS parameters were a probe diameter of 1.3 cm, audio frequency of 1 MHz, duty ratio of 20%, and pulse repetition rate of 1 kHz. Moreover, LIPUS was shown to alleviate retinal degeneration and protect it from degenerative thinning. At the molecular level, it was found that LIPUS treatment induced dynamic transformation of YAP/p-YAP, which was enhanced by caspase-3 blotting, and protected both the retina and RGCs. These results suggest that LIPUS plays a key role in preventing RGCs’ apoptosis through YAP.

Currently, extensive research demonstrated that many other stem cells were also modulated by LIPUS both *in vitro* and *in vivo* (**Table 2**).

**Table 2:** Biological effects of LIPUS on various stem cells.

Cell types	Authors	Year of publication	Physiological effects of LIPUS
GSCs	Song (23)	2022	LIPUS “waked up” GSCs to improve their sensitivity to chemotherapy and induced GSCs differentiation.
RGSs	Zhou (12)	2018	LIPUS protected RGCs from optic nerve injury-induced apoptosis.
BMSCs	Sena (25) Tabuchi (26) Xia (27) An (28) Li (29) Lee (30) Xie (31) Yang (32) Ning (33) Chen (34) Wang (22) He (35)	2011 2020 2017 2018 2018 2008 2019 2019 2019 2019 2014 2019	LIUPS enhanced the proliferation of BMSCs and promoted the differentiation of BMSCs into osteoblasts and chondrocytes, enhanced cell viability and survival rate, and promoted cell migration. The combined application of LIPUS with other substances can also improve the cellular properties of BMSCs.
ADSCs	Huang (36) Wang (37) Jiang (38) Yue (39) Uddin (40) Zhang (41) Fu (42) Nishida (43) Nagasaki (44) Kang (45)	2020 2019 2012 2013 2013 2018 2013 2020 2015 2019	LIPUS promoted the proliferation of ADSCs and promoted the osteogenic differentiation and adipogenesis of ADSCs. And different intensities of LIPUS have different effects on ADSCs. The combined application of LIPUS with other substances can also improve the cellular properties of ADSCs.
PDLSCs	Bialy (46) Hu (47) Li (48) Kusuyama (49)	2012 2014 2020 2017	LIPUS promoted anabolic and osteogenic differentiation of PDLSCs.
DPSCs	Gao (50)	2016	LIPUS promoted the proliferation of DPSCs.
Gingival stem/progenitor cells	Bialy (51)	2014	LIPUS enhances the differentiation of human gingival stem/progenitor cells.
ABMSCs	Lim (52)	2013	LIPUS enhanced the cell viability and osteogenic differentiation of hABMSCs.
NSCs	Wu (53) Lv (54) Xia (55) Lv (56)	2020 2013 2017 2015	LIPUS promoted iPSCs-NCSCs proliferation, cell viability, cytoskeletal morphological changes, neural differentiation of neural stem cells, and regeneration of damaged peripheral nerves.
HSPCs	Xu (57) Liu (58)	2012 2017	LIPUS stimulated the viability, proliferation, differentiation and cell migration of HSPCs, accelerated the construction of bone marrow cells, and increased the quantity and quality of red blood cells, white blood cells and platelets in peripheral blood.
hAD-MSCs	Ling (59, 60)	2017	LIPUS promotes the proliferation of hAD-MSCs
SSCs	Mohaqiq (61)	2018	LIUPS has a good effect on the proliferation and colonization of SSCs.
Ovarian follicular fluid (FL)-derived MSCs	Omes (62)	2013	LIPUS stimulated the proliferation of ovarian follicular fluid-derived MSCs.
s-MPs	Lucas (63)	2021	s-MPs did respond to ultrasound, but showed no changes in cell viability, proliferation, and migration.

## 3 Mechanism of LIPUS in modulating TRSCs

At present, the mechanism of LIPUS regulating TRSCs has been extensively studied, and the relevant cell biology and molecular biology mechanisms have been initially discovered. Related cell signaling pathways were also explored. However, to thoroughly elucidate its mechanism, especially the regulatory mechanism of tissue-resident stem cells, a lot of in-depth basic and clinical research is still needed.

### 3.1 The cellular and molecular mechanisms involved in the interaction

LIPUS has been shown to have a positive effect on TRSCs, promoting their proliferation, differentiation, and migration. The cellular and molecular mechanisms involved in this interaction are complex and not fully understood, but several studies have provided insights into the potential mechanisms underlying the effects of LIPUS on TRSCs.

1) Activation of intracellular signaling pathways: LIPUS has been shown to activate various intracellular signaling pathways, including the MAPK and PI3K/AKT pathways (59), which are involved in cell proliferation, differentiation, and migration. These signaling pathways are thought to play a role in the beneficial effects of LIPUS on TRSCs.
2) Upregulation of growth factors and cytokines: LIPUS has been shown to upregulate the expression of growth factors and cytokines, such as VEGF, bFGF, and TGF-β, which are involved in the regulation of cell proliferation, differentiation, and migration (64, 65). These growth factors and cytokines are thought to stimulate the activity of TRSCs and promote their regenerative potential.
3) Enhancement of extracellular matrix (ECM) remodeling: LIPUS has been shown to promote the remodeling of the ECM (66), which is important for tissue regeneration. TRSCs rely on the ECM to provide physical support and biochemical signals for their proliferation, differentiation, and migration. LIPUS has been shown to stimulate the synthesis and remodeling of ECM components, such as collagen and fibronectin, which are important for tissue regeneration.
4) Induction of angiogenesis: LIPUS has been shown to promote the formation of new blood vessels, which is important for tissue regeneration (45). TRSCs have been shown to play a role in angiogenesis, and LIPUS has been shown to stimulate the production of angiogenic factors, such as VEGF.

Overall, the cellular and molecular mechanisms involved in the interaction between LIPUS and TRSCs are complex and multifactorial. The effects of LIPUS on TRSCs are likely mediated through multiple signaling pathways, growth factors, cytokines, and ECM components, all of which play a role in the regenerative potential of these cells.

### 3.2 Cellular signaling pathways for LIPUS modulate TRSCs

**Figure 2:**
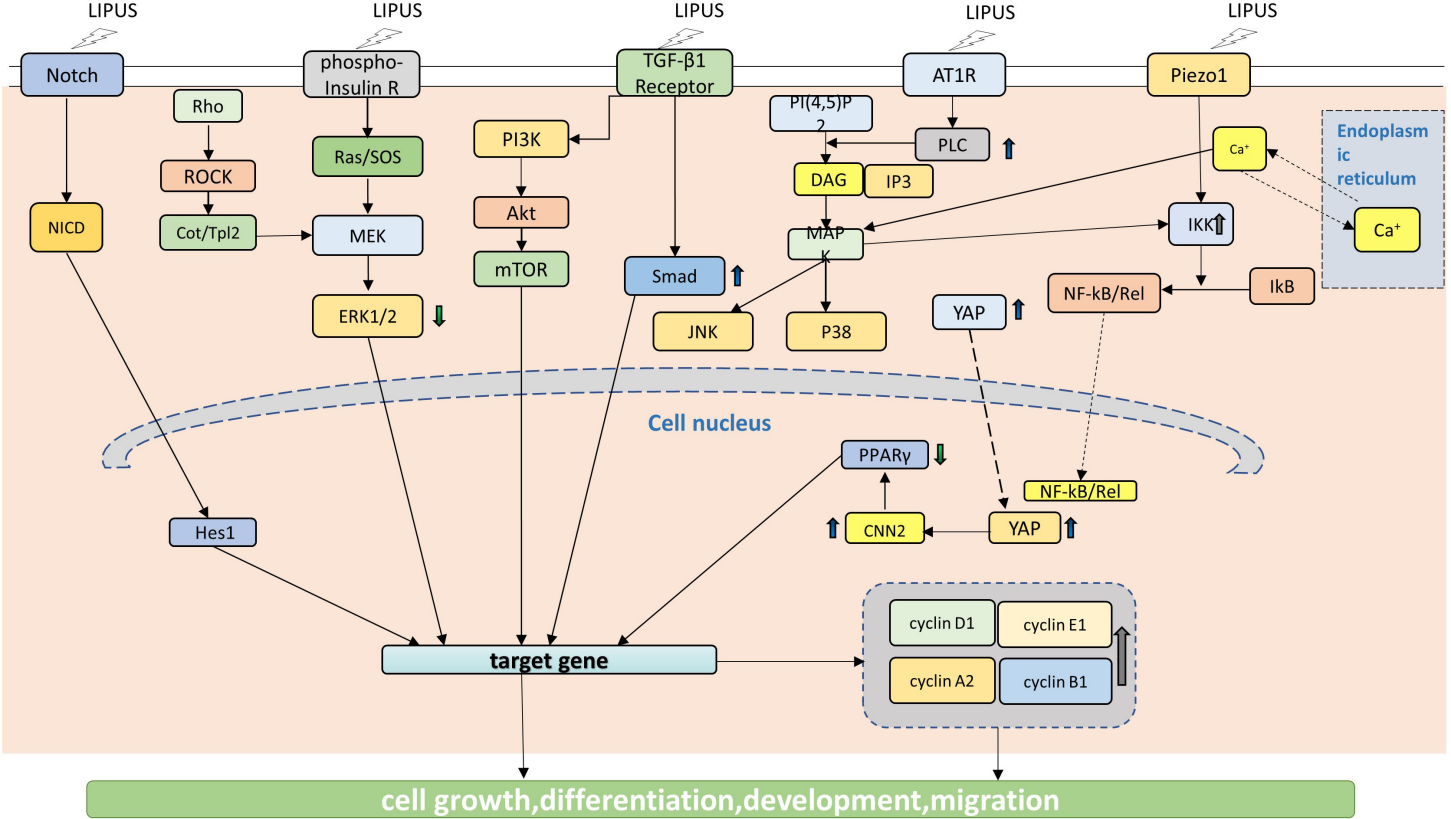
LIPUS acts on signaling pathways in TRSCS. LIPUS triggers the proliferation and differentiation of TRSCs by regulating Notch signaling pathway, which is manifested by the significant up-regulation of Hesl expression level. LIPUS can activate the TGF-B and PI3K/Akt pathways, which are involved in the regulation of cell proliferation, differentiation, and migration. LIPUS also regulates MAPK/ERK pathway, including Rho-Cou/Tpl2-MEK-ERK signaling pathway, Ras/SOS-MEK-ERK signaling pathway, JNK MAPK signaling pathway and p38 MAPK signaling pathway. By activating the TGF-B and PI3K/Akt signaling pathways, LIPUS enhances cell survival and proliferation. Furthermore, LIPUS promotes the translocation of YAP to the nucleus, resulting in the up-regulation of CCN2. The forced expression of CCN2, however, can reduce the expression of PPAR y gene.

Since 2004 (67), researches have been carried out on the cellular signaling pathways mechanically stimulated by LIPUS, especially the pathways related to the stem cells. LIPUS has been shown to modulate the activity of TRSCs through various cellular signaling pathways (**Figure 2**), including **1).** Wnt/β-catenin pathway: The Wnt/β-catenin pathway is involved in the regulation of cell proliferation, differentiation, and migration, and it plays an important role in the development and maintenance of ocular tissues. LIPUS has been shown to activate the Wnt/β-catenin pathway in tissue resident stem cells, leading to enhanced proliferation and differentiation (68, 69). **2).** PI3K/Akt pathway: The PI3K/Akt pathway is a key regulator of cell survival and proliferation, and it plays an important role in the response to growth factors and cytokines. LIPUS has been shown to activate the PI3K/Akt pathway in tissue resident stem cells, leading to enhanced survival and proliferation (31, 59). **3).** MAPK/ERK pathway: The MAPK/ERK pathway is involved in the regulation of cell proliferation, differentiation, and migration, and it plays an important role in the response to extracellular stimuli. LIPUS has been shown to activate the MAPK/ERK pathway in tissue resident stem cells, leading to enhanced proliferation, differentiation, and migration (26, 34, 70, 71). **4).** Notch signaling pathway: The Notch signaling pathway is involved in the regulation of cell fate determination and differentiation, and it plays an important role in the development and maintenance of ocular tissues. LIPUS has been shown to activate the Notch signaling pathway in tissue resident stem cells, leading to enhanced differentiation and regeneration (53). **5).** TGF-β signaling pathway: The TGF-β signaling pathway is involved in the regulation of cell proliferation, differentiation, and migration, and it plays an important role in the response to tissue injury and inflammation. LIPUS has been shown to activate the TGF-β signaling pathway in tissue resident stem cells, leading to enhanced proliferation, differentiation, and migration (27, 41, 72, 73).

At the same time, the YAP signaling pathway, Piezo signaling pathway and SDF-1/CXCR4 signaling pathway are also involved (**Figure 2**) (12, 26, 27, 31, 34, 37, 41, 43, 50, 53, 59, 67, 70–80). These cellular signaling pathways are thought to be involved in the effects of LIPUS on tissue resident stem cells, but the exact mechanisms by which LIPUS activates these pathways are not fully understood. Further research is needed to fully elucidate the cellular and molecular mechanisms underlying the effects of LIPUS on tissue resident stem cells, and to optimize the use of LIPUS for therapeutic applications in ophthalmology.

## 4 Potential applications of LIPUS therapy in ophthalmic diseases

LIPUS has shown great potential for stem cell therapy in various ophthalmic diseases, including 1). Age-related macular degeneration (AMD): AMD is a leading cause of vision loss in older adults. LIPUS can stimulate the proliferation and differentiation of RPE cells, which play a critical role in maintaining the health of the retina. 2). Glaucoma: Glaucoma is a group of eye conditions that can lead to irreversible vision loss. LIPUS can stimulate the differentiation of stem cells into RGCs, which are the cells that are damaged in glaucoma. 3). Corneal injuries and diseases: the cornea is a transparent layer at the front of the eye that can be damaged by injury or disease. LIPUS can stimulate the proliferation and differentiation of corneal epithelial stem cells. 4). Retinal diseases: LIPUS can stimulate the differentiation of stem cells into retinal cells to replace the damaged or destroyed cells. 5). Optic nerve injuries: Optic nerve injuries can result in permanent vision loss. LIPUS can stimulate the differentiation of stem cells into RGCs, which are the cells that are damaged in optic nerve injuries.

## 5 Conclusions

Stem cell research is a highly discussed topic in the field of biological science. Over the years, a growing body of evidence has shown that tissue resident stem cells represent promising targets for regenerative medicine. Sources of stem cells commonly used for retinal regeneration today include endogenous retinal stem cells (such as neural stem cells, Muller cells, and retinal stem cells from the ciliary marginal region) and exogenous stem cells (such as bone mesenchymal stem cells, adipose-derived stem cells, embryonic stem cells, and induced pluripotent stem cells) (81). Leveraging the characteristics of TRSCs, their survival, migration, differentiation and integration in the retina can be utilized to try to restore vision in patients with retinal diseases (82). LIPUS, a non-invasive therapeutic method, has been demonstrated to effectively modulate RGCs (12). However, the system specifications of LIPUS for stem cells must be carefully optimized to ensure that the desired effects are achieved without causing any harm to the tissue or the stem cells themselves. In the future, LIPUS is anticipated to be applied to treat glaucoma, retinal degeneration, and other optic nerve injury diseases. The focus of future LIPUS research in the field of ophthalmology will be on how to enhance its efficiency and accuracy, as well as understanding the underlying biological mechanisms involved.

## Author contributions

AD and ZL contributed to conception and design of the study. ZL wrote the first draft of the manuscript. NH, LG and XZ wrote sections of the manuscript. YZ and ZC participated in data analysis and interpretation. All authors contributed to the article and approved the submitted version.
